# Protecting Vulnerable Research Subjects in Critical Care Trials: Enhancing the Informed Consent Process and Recommendations for Safeguards

**DOI:** 10.1186/2110-5820-1-8

**Published:** 2011-04-13

**Authors:** Henry Silverman

**Affiliations:** 1 University of Maryland School of Medicine, 110 South Paca Street; 2nd floor, Baltimore, Maryland, USA 21201, USA

## Abstract

Although critically ill patients represent a vulnerable group of individuals, guidelines in research ethics assert that ethically acceptable research may proceed with such vulnerable subjects if additional safeguards are in place to minimize the risk of harm and exploitation. Such safeguards include the proper obtainment of informed consent that avoids the presence of the therapeutic misconception and the assessment of decisional capacity in critically ill patients recruited for research. Also discussed in this review are additional safeguards for such vulnerable subjects, as well as the issues involved with proxy consent. Heightened awareness to principles of ethics and provision of additional safeguards to enhance protections of vulnerable subjects would help to maintain the public trust in the research endeavor.

## Introduction

Critical care research presents special ethical challenges largely due to the potential for exploitation stemming from the vulnerability of the critically ill patients who are solicited for enrollment. Vulnerability refers to the inability to protect oneself and can be due to intrinsic (e.g., deficits in decision-making capacity) and situational factors that threaten voluntary choice (e.g., coercive settings or undue inducements) [1,2]. The presence of vulnerability makes the achievement of a valid, informed consent problematic. Nonetheless, consensus statements on research ethics assert that ethically acceptable research may proceed with such vulnerable subjects if additional safeguards are in place to minimize the risk of harm and exploitation [2-5].

However, research involving critically ill patients remains problematic due to the lack of guidance on how to protect vulnerable subjects adequately [1,6]. For example, the U.S. Common Rule's general provisions merely direct Institutional Review Boards (IRBs) to include "additional safeguards...to protect the rights and welfare" of "mentally disabled persons" [7]. Similar lack of guidance as well as ambiguity has been raised with the European Union Directive [8,9]. In the absence of the specification of at least the essential safeguards, the protection of vulnerable subjects relies too heavily on the views of diverse IRBs, leading to insufficient protection of vulnerable subjects [10]. For example, in its investigation of critical care trials, the Office for Human Research Protections (OHRP) found that most IRBs failed to require additional safeguards beyond that of requiring proxy consent [11]. In a cross-sectional survey of IRBs in U.S. institutions, investigators showed that IRBs rarely or never required procedures to determine capacity and also varied in their use of other safeguards, such as independent monitors, research proxies, and advance research directives [10].

This article is designed to review a) the issues involved with the decision-making capacity of critically ill patients, b) the methods of capacity assessment, and c) the potential concerns regarding proxy consent. A framework is also presented to outline specific core safeguards linked to risk levels that will minimize the possibility of exploitation of critically ill patients who are asked to enroll in clinical trials.

## Informed consent and the capacity to consent

Informed consent provides research subjects a mechanism to protect themselves. Such a protection mechanism is necessary, because unlike in the clinical setting in which the interests of patients and doctors converge, researchers' interests in obtaining valid scientific data can conflict with their obligation to protect the rights and welfare of the research participants.

Critical care investigators face a difficult task of recruiting research subjects who may have diminished capacity to understand information, appreciate the situation they are in, and to make complex decisions. Indeed, studies have shown that patients with acute illnesses may have limitations in their decision-making capabilities due to a number of factors, including the presence of delirium, their underlying illness, or the use of sedatives and analgesics [11-16]. The presence of these factors does not necessarily translate into incapacity to provide a valid informed consent to research. Indeed, two types of errors can occur in the setting of obtaining informed consent from critically ill patients. One, surrogate consent is obtained for patients who are capable of providing consent, thus depriving such patients their autonomy rights to choose for themselves. The opposite error occurs when investigators obtain informed consent from patients who lack decisional capacity, thus making them vulnerable to exploitation. Several studies have documented that many enrolled subjects have limited understanding of the research to which they provided consent [17-20].

Another issue with the proper obtainment of informed consent involves the ability of research participants to distinguish between research and clinical care [21]. "Therapeutic misconception" (TM) is used to describe this phenomenon, a term first reported by Appelbaum and colleagues in 1982 during interviews with patients with psychiatric disorders who participated in clinical trials [22]. Indeed, several empirical studies have shown that individuals participating in clinical research misconstrue a therapeutic intention to the research procedures in a study [23-26].

Investigators have identified two ways in which TM can be manifested: 1) when research participants fail to recognize that decisions regarding randomization or certain aspects of the research procedures (e.g., dosages and duration of administered drugs) will not be individualized to their personal needs (TM1); or 2) when research participants hold an unreasonable appraisal of the nature or likelihood of medical benefit from their study participation (TM2) [23]. Essentially, research participants might hold mistaken beliefs about how the research will be executed (TM1) or is designed in a manner to ensure direct benefits to them (TM2) [22].

Investigators have explored the frequency and the factors (both participant- and study-related) that might underlie the existence of TM in clinical research [23,25-27]. In a study involving patients in different types of clinical trials, those with greater age, lower levels of education, poorer health and functional status, and those with greater optimism about their health in the future were at higher risk for manifesting TM [23]. In another study involving early-phase gene transfer trials, participants with cancer or vascular diseases exhibited TM more frequently than those with inherited or infectious diseases. Also, the presence of TM was significantly less in patients who received a consistent message that benefits were unlikely compared with those who did not receive any such messages [26]. Finally, in a study involving more than 270 patients enrolled in clinical trials, TM was present in 70% of respondents and it presence was positively associated with the acuteness and severity of the disease [27].

The presence of TM might be explained by participants' knowledge gap regarding how clinical research and patient care differ in their purpose, characteristic methods, and justification of risks [21]. Alternatively, it might be due to participants' misplaced trust in researchers, thinking that they will act as their physicians who will protect them, as well as promote their individual health interests [28]. Finally, the conduction of research studies in the clinical setting might reinforce the presence of treatment relationships and individualized care. Accordingly, many patients who enroll in research might think that their care will still be individualized to match their personal medical needs even in the context of a research study [29]. Such perceptions might be enhanced by the use of certain language found in consent forms (such as "doctor" instead of "investigator," and "treatment" instead of "intervention"), the "therapeutic" discourse used by nurses and physicians conducting clinical trials [30], or the recruitment of patients into clinical trials by investigators who also their primary physicians.

There are two major ethical concerns with the TM. First, failure to appreciate correctly the risks and benefits of research participation raises concerns regarding the validity of informed consent [21,23]. Second, the presence of TM reflects the very real possibility that research participants will see the investigator's role as that of the physician and view an invitation to enroll in research as a professional recommendation that is intended to serve their individual treatment interests. Such inappropriate enrollment of patients into research reflects a concern with exploitation [31].

## Methods to enhance informed consent for participation in clinical trials

The written, informed consent form (ICF) can be instrumental in enhancing subject comprehension. A study involving the informed consent process in cancer clinical trials showed that careful reading of the consent form was associated with improved knowledge scores [25]. Other studies have demonstrated greater comprehension in those subjects who have carefully read the consent form [32,33], but these findings were associated with individuals with higher education levels. This result is not surprising considering that many IRB-approved ICFs are written at a reading level that is above that of the general population [34-36].

To enhance subject understanding, commentators have recommended the use of shorter, simplified forms containing language at or below the eighth-grade reading level [37,38]. Studies have demonstrated that certain modifications in the written ICF could lead to enhanced subject comprehension. Dresden and Levitt [39] showed that subjects were able to retain more information from a modified, shortened version (reading level = 8.7) than from a standard, industry consent form (reading level = 12.0). Young et al. [40] reported significantly higher subject comprehension using a simplified form (sixth-grade level) compared with a standard form (16th grade level). Another study showed that self-reported understanding by patients tended to be highest when consent forms were written at a lower grade level [33]. These results suggest that shorter ICFs written at a lower reading level, when read carefully, might enhance subject understanding. Recently, the Acute Respiratory Distress Syndrome Clinical Trials Network (ARDSNet) investigators published an informed consent template exclusively for critical care research that is written at an eighth-grade reading level, highlights key research concepts, and uses language to minimize the therapeutic misconception (e.g., avoidance of words, such as "treatment," "therapy," or "medication," which might imply that the agent or procedure has some proven efficacy; and use of "investigator" or "study doctor" instead of the word "doctor") [41].

In addition to the format and structure of the consent forms, the use of alternative media or methods for presenting information might enhance comprehension. A recent review analyzed the existing literature on interventions to improve research participants' understanding of information disclosed in the informed consent process [42]. The analysis reviewed five categories of interventions: multimedia presentations, enhanced consent forms, extended discussions, test/feedback, and "other." The results showed that the use of multimedia and enhanced consent forms had only limited success, whereas having extended discussions appeared to be the most effective method of improving understanding.

## Methods to assess capacity

Practices regarding the obtainment of informed consent from critically ill patients differ among researchers. For example, in the ARDSNet trials involving critically ill patients receiving mechanical ventilation (Low Tidal Volume Trial and the Fluids and Catheter Treatment Trial), approximately 1% of research subjects were deemed competent to provide their own consent. However, there was considerable variability between the clinical centers with regards to the proportion of critically ill patients who gave informed consent themselves (range between 0% and 16.3%) [11]. This variability coupled with the lack of a specific plan to assess decision-making capacity by many of the ARDSNet clinical centers might question the validity of the informed consent process. The failure to assess capacity can be problematic, because incorrect judgments that potential vulnerable subjects are capable of exercising autonomy might involve them in research involuntarily. Accordingly, commentators have suggested that researchers assess subjects' comprehension as part of the informed consent process [43-45].

Currently, there are no universally accepted procedures regarding capacity assessment for critical ill patients. However, there are several well-validated measures for evaluating informed consent capacity for decision making in the research setting [46]. Many of these instruments are time-consuming in its application, thus making it somewhat undesirable for use as a screening measure in most research studies with critically ill patients. Alternatively, a shorter measure is the University of California, San Diego (UCSD), Brief Assessment of Capacity to Consent (UBACC) instrument, which is a 10-item measure that uses questions pertaining to understanding and appreciation of information [47].

Less formal capacity assessments can consist of using probing questions to assess the potential subjects' understanding of the involved research. Johnson-Greene [44] recommend the following examples of probing questions that researchers could ask after describing the research:

1. Can you tell me what will happen if you decide to be in this study?

2. Will being part of this study help you?

3. Can anything bad happen to you if you are part of this study?

4. Can you decide not to be part of this study?

Also recommended is that when asking probing questions to assess capacity, researchers should avoid the use of leading questions or close-ended questions that elicit a "Yes" or "No" response. This recommendation resonates with the previous-mentioned finding that comprehension is enhanced when investigators have extended discussions with potential research participants. In other words, being able to have conversations with potential participants is crucial in explaining the research protocol and assessing their capacity. Since having such conversations with patients receiving mechanical ventilation is difficult, the feasibility of enrolling such patients with their own informed consent might be problematic, especially if these patients are receiving or have received sedatives. One approach to patients who are receiving mechanical ventilation is to assess whether such patients have some decisional capacity and, if present, obtain their assent in conjunction with the consent of their proxies (similar to what is done with children).

## Issues involved with proxy consent

Although many critically ill patients have, or are at risk of having, decisional impairment, consensus statements on research ethics assert that ethically acceptable research may proceed with such vulnerable subjects with appropriate proxy consent [2,3]. Both the United States federal regulations [7] and the European Directive [8,9] require proxies who give consent for subjects' participation in research to be *legally authorized *under applicable law to provide such consent.

Who shall serve as the legal authorized representative (LAR) for the patient is determined by the laws of the respective state in the United States or by the national law of the individual European Union member nation. LARs might include persons previously appointed through a legal process (e.g., legal guardian). Alternatively, laws might confer automatic legal authorization to existing "natural persons" (such as a family member or a friend). Without laws that give such automatic legal authorization to family or friends, many previously healthy persons who become temporarily incapacitated due to critical illness may not be able to participate in many types of critical care research, because they had not previously appointed a legal representative.

Whether state laws were in existence that gave legal authorization to family members to provide consent was a recent focus of OHRP in the ARDSNet trials [45]. In European countries with laws that define a narrow interpretation of "legal representatives," researchers have reported extensive difficulties with enrolling patients in research [9,48].

There are two decision-making standards for proxy consent. If possible, proxies should appeal to the "substituted judgment" standard, whereby decisions for incapacitated patients are based on a good faith judgment of what subjects would have chosen if capable of making a decision themselves. However, such a standard is frequently unrealistic because proxies often do not know patients' previous preferences [49,50]. Accordingly, proxies should consider what would be in the "best interests" of the patient. Regardless of the decision-making standard used to provide consent, proxies should be appropriately qualified (e.g., properly acquainted with the patient and available to be present during the course of the research so that they will be ready to revoke consent if the burdens become too great) and not subject to potential conflicts of interest [9]. Also, studies have shown high levels of anxiety and psychological distress in family members of critically ill patients, which might impair their ability to give adequate informed consent for research participation for incapacitated patients [51,52].

## Core safeguards for vulnerable subjects

Although the U.S. regulations are silent regarding specific safeguards to provide additional protections to adults unable to provide consent, other guidelines have proposed the following safeguards: 1) assessment of subjects' decision-making capacity; 2) respect for subjects' assent and dissent; 3) a process to obtain re-consent if and when the subjects regain capacity; 4) "necessity" requirement to ensure that research cannot be performed without enrolling incapacitated adults; 5) "subject-condition" requirement, whereby the research involves a condition from which the subject suffers; 6) an independent participation monitor to monitor subjects' involvement as they progress through the study protocol; 7) an independent consent monitor to monitor the consent process and assess subject's capacity; and 8) sufficient evidence of subjects' prior preferences and interests [1,5,53,54].

One study surveyed the practices of 104 IRBs from a random sample of U.S. institutions and demonstrated much variability regarding the requirement of safeguards and limits on risks for research involving adults with impaired decision-making capacity [10]. For example, the survey results showed the percentage of IRBs that would "always" or "very frequently" require the following safeguards: determination of consent capacity for patients in the intensive care unit (56%); presence of assent or dissent from patients (50%); presence of an independent monitor to provide assurance that research participation is consistent with the patient's interest (13%); re-consent of the subjects once decision-making capacity returns (75%); and evidence of the patient's previous preference for research (27%).

Regarding allowable risk-to-benefit ratios, this survey study showed that for research with potential for direct benefit, 62% of IRBs had no limits on acceptable risks and 24% would limit risks to no more than a minor increment over minimal risk. International guidelines also differ on the allowable risk levels for studies involving adults who lack decision-making capacity [55]. For research with no prospect of direct benefit, 14% of IRBs would not allow research of any risk to involve incapacitated adults, whereas 39% would allow only minimal risk research, and 25% would allow research with minimal risk and those with a minor increase over minimal risk.

Commentators have argued that for research involving vulnerable subjects, the risk of research procedures that do not offer a prospect of direct benefit should be capped at the level of minimal risk [5,56,57]. This position reflects the concern that vulnerable subjects should not be put at undue risk for the sake of society and that such research is exploitative. However, advocating such a risk ceiling would seriously impair important research. Also, there is justification for allowing research procedures without a prospect of direct benefit and no more than a minor increment above minimal risk. For example, with a concept of minimal risk reflecting an absolute standard linked to socially acceptable risks, procedures that involve a minor increment above minimal risk would pose no significant threat to the subject's health. Procedures common in critical care research relevant to this risk category would include non-contrast CT scans and the obtainment of additional bronchoalveolar lung (BAL) fluid in patients undergoing this procedure for clinical diagnostic purposes.

Commentators have recommended a framework delineating safeguards linked to permissible risk levels of the proposed protocol procedures [45]. Table 1 shows a proposed hierarchy of risk levels and associated safeguards. The risk levels and benefit factors presented refer to the individual procedures of the study, rather than the research study as a whole. Indeed, a research study may consist of several procedures or distinct components: those with and those without a prospect of direct benefits. The latter would include those done solely to gather data to answer a research question. In the absence of such a component analysis, procedures performed solely for research purposes might claim to be justified by the procedures that offer the prospect of direct benefits to subjects [58]. Because any given protocol may contain many procedures and hence, a range of risk levels, the selection of the essential safeguards to protect vulnerable subjects should be based on highest risk level present in the protocol.

**Table 1:** Risk levels and essential safeguards for research studies involving critically ill patients

Risk level	Proposed safeguards
Level I: Procedures do not involve greater than minimal risk for any research.	• A written plan describing methods to assess decision making capacity If subjects do not have capacity to provide consent, then: • Proxy consent; • A process to respect assent and dissent of the subjects; • A process to obtain re-consent from the subjects if and when subjects regain capacity
	
Level II: Procedures of the research involve greater than minimal risk and offer the prospect of direct benefits.	• Level I safeguards • Participation monitor: availability of an independent person to monitor the subject's involvement in the study, e.g., the subject's legal authorized representative.
	
Level III: Procedures of the research do not involve greater than a minor increment above minimal risk and do not offer the prospect of direct benefits.	• Level I and II safeguards • Necessity requirement • Subject condition requirement
	
Level IV: Procedures of the research involve greater than minimal risk and do not offer the prospect of direct benefits.	• Level I, II, and III safeguards • Independent consent monitor • Evidence of the subject's preferences and interests.

For research involving vulnerable subjects at all risk levels, the following safeguards are recommended:

1) Investigators outline a specific plan to assess the capacity of all potential subjects when groups that might involve persons with decisional impairment are targeted for research, for example, critically ill patients;

2) Proxy consent for those subjects found to lack decision making capacity;

3) For subjects found to lack decision making capacity to consent, there should be a requirement for assent, which entails that investigators obtain affirmative agreement to research participation from subjects whose capacity is considerably but not completely diminished. Such subjects might still be able to understand some aspects of a study. In a similar construct, expressions of dissent should be respected; and

4) A process for re-consent, which entails that subjects who were previously impaired and who regain capacity should be asked for their personal consent, regardless of whether the procedures in the research are completed.

For research with procedures involving more than minimal risk and offer the prospect of direct benefits, an additional safeguard would be to ensure the availability of an independent person to monitor the subject's involvement in the study, mainly to determine when it might be appropriate to withdraw the subject from the study. The subject's legally authorized representative could ordinarily fulfill this role of a participation monitor.

For research with procedures involving no more than a minor increment above minimal risk and do not offer the prospect of direct benefits, additional safeguards for this risk level include:

1) The "necessity requirement," which entails that subjects with decisional impairment should be enrolled in research only when their participation is scientifically necessary, for example, when the desired information cannot be obtained by enrolling adults who can consent; and

2) Subject condition requirement, whereby the research must involve a condition from which the subject suffers.

The most controversial category of research are studies containing procedures with no prospect of direct benefits and present more than a minor increment above minimal risk. Due to the potential for serious harm and exploitation, an independent consent monitor should be available who could assess decision-making capacity and assess evidence of the subject's prior preferences and interests to participate in research.

## Conclusions

The ethical conduct of research is complex, evolving, and harbor potential harms and infringement of rights to subjects who participate in such research. This is especially true for research involving critically ill patients, many of whom are vulnerable due to potential decisional incapacity and the setting of the research itself. Heightened awareness to principles of ethics and incorporation of procedures to enhance the informed consent process and the provision of additional safeguards to enhance protections of vulnerable subjects would help maintain the public trust in the research endeavor.

## Competing interests

The authors declare that they have no competing interests.

## Author information

Henry Silverman is Professor of Medicine at the University of Maryland School of Medicine, USA. He is chair of the hospital's Clinical Ethics Committee and is Program Director of the Middle East Research Ethics Training Initiative (MERETI) sponsored by the Fogarty International Center at the National Institute of Health (see http://www.mereti.net).
